# Sexual dimorphism in gastric cancer: tumor-associated neutrophils predict patient outcome only for women

**DOI:** 10.1007/s00432-019-03082-z

**Published:** 2019-11-19

**Authors:** Franziska Clausen, Hans-Michael Behrens, Sandra Krüger, Christoph Röcken

**Affiliations:** Department of Pathology, Christian-Albrechts-University, Arnold-Heller-Str. 3, Haus U33, 24105 Kiel, Germany

**Keywords:** Gastric cancer, Tumor immune microenvironment, Tumor-associated neutrophils, Sexual dimorphism

## Abstract

**Purpose:**

Tumor-associated neutrophils (TANs) are part of the tumor immune microenvironment (TIME) and may contribute to gastric cancer (GC) biology. We hypothesized that TAN are enriched in the TIME, show sex-specific differences, and correlate with patient outcome.

**Methods:**

We analyzed the distribution and putative tumor biological significance of TANs in a well-characterized, therapy-naïve, European GC cohort using immunohistochemical staining of myeloperoxidase (MPO), and digital image analysis using Definiens Tissue Studio^®^.

**Results:**

Different tumor compartments were examined, and TAN densities were correlated with various clinicopathological patient characteristics. TAN density showed a large interindividual variability ranging from 0 to 6711.0 TANs/mm^2^. Intratumoral distribution patterns were inhomogeneous (tumor surface vs. tumor center vs. invasion front) and correlated significantly with Laurén phenotype, tumor grade, and microsatellite status in the tumor center and invasion front. In the multivariate analysis, TAN density in the invasion front was an independent predictor of tumor-specific survival only for women (HR = 2.77, *p*   < 0.001). In men, no correlation was found between TAN density and survival.

**Conclusion:**

With regard to TANs, our study independently validates sexual dimorphism in GC biology.

**Electronic supplementary material:**

The online version of this article (10.1007/s00432-019-03082-z) contains supplementary material, which is available to authorized users.

## Introduction

Gastric cancer (GC) is the fifth most common cancer worldwide and the third most common cause of cancer death. Incidence and mortality rates are steadily declining, except for cancers of the cardia and gastroesophageal junction, whose incidence has been stable or even increasing. GC is generally more common in men, and incidence increases with patient age (ENCR 2017; Stewart and Wild 2014). Major risk factors are infection with *H. pylori* and dietary habits. In addition, a minority of GC is also linked to Epstein-Barr virus (EBV) infection (Humans 2012). Family history and gene polymorphisms modulate individual cancer risk (Saeki et al. 2013).

Neutrophils are an essential part of the innate immune system and provide protection against microbial infections. Tumor-associated neutrophils (TANs) can be found as part of the tumor immune microenvironment (TIME) (Binnewies et al. 2018). They are recruited to the tumor site by a chemotactic gradient involving different cytokines, chemokines, and growth factors, provided by the tumor itself or recruited cells. TANs have been associated with a poor prognosis in cancers, most notably in hepatocellular carcinoma, intrahepatic cholangiocarcinoma, head and neck cancer, renal cell carcinoma and non-small-cell lung cancer (Shen et al. 2014). However, a dual role of TANs in cancer biology has been described since they can both promote and inhibit cancer progression. Tumor-suppressing N1-TANs show direct and antibody-dependent cell-mediated cytotoxicity against tumor cells. They also produce proinflammatory cytokines and recruit CD8^+^T cells (Shaul and Fridlender 2018). N2-TANs are considered tumor promoting. They promote tumor extravasation and angiogenesis, suppress the immune system, and recruit CD4^+^T cells, and so promote tumor growth and metastasis (Shaul and Fridlender 2018). IFN-γ might stimulate neutrophils to upregulate programmed cell death 1 ligand 1 (PD-L1) and suppress T cell proliferation. Both N1- and N2-TANs are considered mature neutrophils (Shaul and Fridlender 2018). Factors produced by tumors and stroma have been postulated to influence TAN differentiation (Fridlender et al. 2009; Piccard et al. 2012). Collectively, these data provide evidence that TANs, in general, play an active role in cancer biology.

In the past, a limited number of studies provided evidence that TANs may also be involved in GC biology, showing divergent results with regard to the protective (Abe et al. 2016; Caruso et al. 2002; Huang et al. 2018; Zhang et al. 2018) or promoting effects (Li et al. 2017; Wang et al. 2017; Zhao et al. 2012) of TANs. Only a single study was carried out on Western patients, which, interestingly, showed evidence of a sex-dependent effect (Caruso et al. 2002) pointing towards sexual dimorphism. Indeed, GC shows a striking sex-specific difference in its susceptibility. According to the European Network of Cancer Registries, the estimated GC incidence in men is almost double that of women. A similar picture was observed for mortality, with an estimated 63,600 stomach cancer deaths in men and 43,700 in women (ENCR 2017). These differences cannot be explained by *H. pylori* infection, the major risk factor for GC (Brusselaers et al. 2017; Group 2001). However, the immune response shows sex-specific differences with regard to infectious diseases, vaccination and autoimmunity, and the effector functions of immune cells are influenced by estrogen and androgen exposure (Markle and Fish 2014). This sexual dimorphism in immune response capacity is now well recognized, and differences in immune surveillance competence between men and women may also contribute to the sex effect observed in malignant tumors (Dorak and Karpuzoglu 2012). However, sexual dimorphism in biomedical science is often not specifically addressed and many studies fail to analyze results by sex (Beery and Zucker 2011).

To fill this gap of information, and in order to shed further light on TIME and sex-specific differences in GC, we aimed to test the following hypotheses in an European GC cohort: (a) TANs in GC are enriched in the TIME; (b) TANs show sex-specific differences; and (c) TAN densities correlate with patient outcome.

## Materials and methods

### Ethics

All executed procedures were in accordance with the ethical standards of the responsible committee on human experimentation (institutional and national) and with the Helsinki Declaration of 1964 and later versions. Ethical approval was obtained from the local ethical review board (D 453/10 and D 468/17). All patient data were pseudonymized prior to study inclusion. All experimental work complied with all mandatory laboratory health and safety procedures.

### Study population

We retrospectively examined all patients who had undergone either total or partial gastrectomy for adenocarcinoma of the stomach or the gastroesophageal junction between 1997 and 2009. Specimens were obtained from the archive of the Institute of Pathology, University Hospital Kiel. The following patient characteristics were retrieved from the electronic database: tumor location, type of surgery, age at diagnosis, sex, tumor type, tumor grade, residual tumor status, tumor size, depth of invasion, number of lymph nodes resected and number of lymph nodes with metastases. Patients were included if a primary adenocarcinoma of the stomach or gastroesophageal junction was histologically confirmed. Patients were excluded if (1) histology identified a tumor type other than adenocarcinoma, (2) patients had undergone a perioperative chemo- or radiotherapy, (3) basic clinicopathological variables (i.e. sex, Laurén phenotype, T-, N-, and M-category, or UICC stage) or (4) more than two of the study compartments (i.e. mucosa, tumor surface, tumor center, and invasion front) were missing (Suppl. Figure 1). Date of patient death was obtained from the “Epidemiological Cancer Registry” of the state of Schleswig–Holstein, Germany. Follow-up data of those patients who were still alive were retrieved from hospital records and general practitioners.

### Histology

Specimens had been fixed in formalin and embedded in paraffin (FFPE). Paraffin sections were stained with hematoxylin and eosin. Tumors were classified according to Laurén (Lauren 1965). The pathological tumor (T), node (N), distant metastasis (M) stage of all study patients was defined according to the 8th edition of the International Union Against Cancer guidelines (Brierley et al. 2016).

### Myeloperoxidase immunohistochemistry

Immunostaining was carried out using the Bondmax automated slide staining system (Leica Microsystems, Wetzlar, Germany) and a rabbit polyclonal anti-human-MPO antibody (dilution 1:2000; Dako, Carpinteria, CA, USA) diluted with Bond Primary Antibody Diluent (Leica, Newcastle, GB). Immunostaining was visualized with the Bond Polymer Refine Detection Kit (Leica Biosystems, Newcastle, GB). Automated antigen retrieval was carried out using Bond Epitope Retrieval Solution (Leica Biosystems, Newcastle, GB) at 20 min. Omission of the primary antibody served as a negative control.

### Image analysis and virtual microscopy

**Fig. 1:**
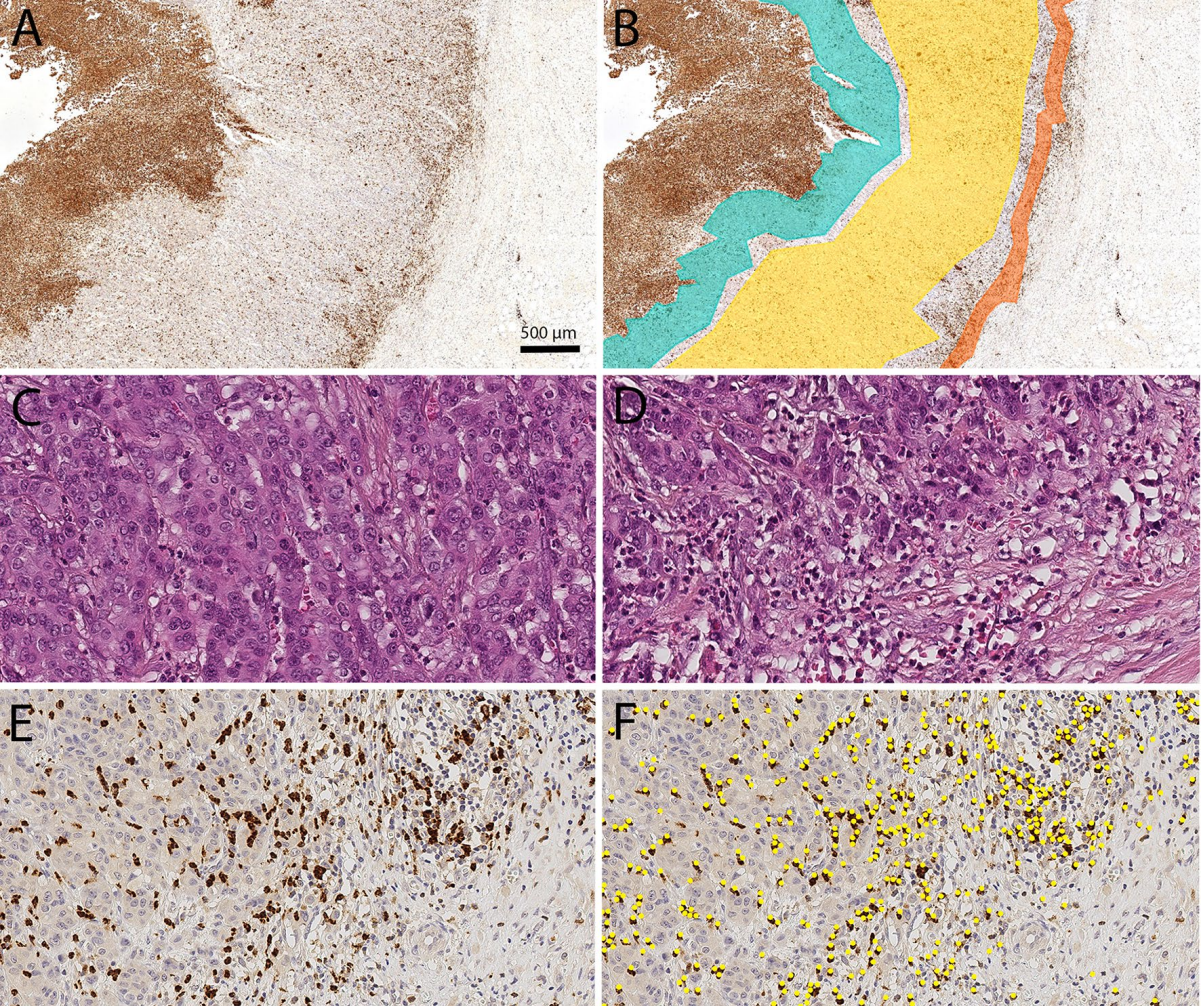
Digital image analysis: Digital image analysis was used to quantify the spatial distribution of tumor-associated neutrophils (TANs) in gastric cancer. The viewer and painting program VMP was used to mark the tumor compartments: tumor surface (green), tumor center (yellow) and invasion front (orange; **a**, **b**). The TAN density was lower in the tumor center (**c**) than in the invasion front (**d–f**). TAN density was quantified by image analysis using Definiens Tissue Studio^**®**^ (TANs identified by Definiens are marked as yellow dots; **f**). All figures were captured from the same tumor. Anti-myeloperoxidase immunostaining (**a**, **b**, **e**, **f**). Hematoxylin and eosin (**c**, **d**). Overview (**a**, **b**). Original magnification 400-fold (**c–f**)

Digital images of immunohistochemically stained tissue sections were obtained using a Leica SCN400 microscopic whole-slide scanner (Leica Biosystems, Nussloch, Germany) at its maximum, nominally 40-times magnification. The pixel-to-pixel distance equals 0.25 µm in the virtual image. The scanned images were exported from the scanner system as Leica SCN format files. To detect MPO^+^ cells, semiautomatic image analysis with Definiens Tissue Studio (version 3.6.1, Definiens, München, Germany) was performed at 20-times magnification. Software settings were used to vary the programmed outputs and classify the cells wanted. Tissue-background separation distinguishes between tissue and background (auto threshold; multiple tissue pieces; 10,000 µm^2^ minimum tissue size). Nuclei were detected (nucleus detection: 0.1 hematoxylin threshold; 50 µm^2^ typical nucleus size), and virtual cell borders were designated (cell simulation: simulation mode = grow from nuclei; 1 µm maximum cell growth). Depending on the intensity of brown chromogen (general settings: stain combination = HC brown chromogen; IHC marker = cytoplasm), the designated cell was classified as a TAN (cell classification: selected feature = HC marker intensity; measurement in = cell; threshold none/low  = 0.5). Analyses were exported as CSV files, which had to be converted in order to be used. The viewer and painting program VMP was used to mark four tumor compartments (Fig. 1).

### Marking compartments

Four tumor compartments were identified: nonneoplastic peritumoral mucosa, tumor surface, tumor center and invasion front. The peritumoral mucosa consisted of the whole tumor-free area between the muscularis mucosa and the mucin layer. For the tumor surface compartment, we marked an area from the surface up to 500 μm into the tumor, avoiding the necrotic layers directly covering the luminal tumor surface. In the tumor center compartment, many parts of the tumor were captured. The marked invasion front was up to 250 μm wide and could include small parts of the surrounding stroma. All marked compartments avoided tissue-free space (e.g., artifacts generated during the cutting of the FFPE tissue samples), large necrotic areas or neoplastic glands wider than 200 μm filled with neutrophil debris and apoptotic bodies.

### Assessment of phenotypic and genotypic characteristics of the study cohort

*H. pylori* (Warneke et al. 2013a, b), EBV (Warneke et al. 2013a, b), HER2 (Warneke et al. 2013a, b), MET (Metzger et al. 2016), and MSI (Mathiak et al. 2017) statuses were assessed as previously described.

### Study design

Whole tissue sections from GCs were stained with an antibody directed against MPO. The density of MPO^+^TANs within the tumor compartments, the tumor surface, tumor center, invasion front, and peritumoral area was calculated. The results were correlated with clinicopathological patient characteristics and survival. The study was carried out following REMARK criteria (Altman et al. 2012).

### Statistical analysis

Statistical analyses were performed using SPSS 24.0 and 25.0 (IBM Corporation, New York, USA). TAN densities were first examined as raw score values by the Wilcoxon signed-rank test, then dichotomized at the median and divided into four groups by splitting the densities into quartiles (Q1, Q2, Q3 and Q4). Subsequently, quartiles were grouped into a TAN-low (Q1) and a TAN-high (Q2, Q3, Q4) group. The significance of the correlations between clinicopathological variables was tested using Fisher’s exact test. For ordinal scale variables, we used Kendall’s tau test for the calculation instead. A p-value was considered statistically significant if *p* ≤ 0.05. To compensate false discovery rate within the correlations we applied the Simes (Benjamini-Hochberg) procedure (multiple testing correction). Overall (OS) and tumor-specific survival (TSS) were computed using the Kaplan–Meier method and compared by log-rank test to determine the significance of differences between the survival curves. The cohort was also separated by sex, and raw score values, OS, and TSS were calculated again. To estimate their impact, clinicopathological features and genetic alterations were correlated by sex and their impact on survival. Additionally, a multivariate Cox regression model was carried out by counting all factors with *p* ≤ 0.10 in the univariate analysis. Sample size calculation for survival analysis was done according to Schoenfeld (1983). We assumed a type II error rate *β* = 0.2.

## Results

In total, 449 patients fulfilled all study criteria. The median age at the time of diagnosis was 68 years (range 28–92 years). A total of 285 (63.5%) patients were males and 164 (36.5%) were females. 239 GCs had an intestinal phenotype, 136 had a diffuse phenotype, 29 were mixed, and 45 were unclassifiable. Overall survival data were available in 433 (96.4%) cases, and tumor-specific survival data were available in 405 cases (90.2%).

### TAN density as a function of the tissue compartment

First, we examined the distribution of TANs in four different compartments, i.e., the peritumoral nonneoplastic mucosa, tumor surface, tumor center, and invasion front (Fig. 1). The median density (n/mm^2^) of TANs differed significantly between the four different compartments (*p* < 0.001). The highest median density was found at the tumor surface (870.2 TANs/mm^2^) and the lowest in the nonneoplastic mucosa (57.9 TANs/mm^2^; Table 1). Interestingly, the median number of TANs was lower in the tumor center (130.0 TANs/mm^2^) than in the tumor surface (870.2 TANs/mm^2^) and invasion front (226.8 TANs/mm^2^; Table 1). These data provide evidence that TANs in GC are not simply related to an unspecific inflammatory response, e.g., loss of mucosal barrier function, but are specifically spatially enriched at the invasion front.

**Table 1:** Densities of myeloperoxidase-immunoreactive tumor-associated neutrophils in four different compartments of gastric cancer

TAN-Density [n/mm^2^]	Mucosa					Tumor surface					Tumor center					Invasion front				
	*n*	25%-P.	Median	75%-P.	Range	*n*	25%-P.	Median	75%-P.	Range	*n*	25%-P.	Median	75%-P.	Range	*n*	25%-P.	Median	75%-P.	Range
Total	257	24.9	57.9	121.1	2.0 – 2022.4	358	483.5	870.2	1430.6	5.8 – 4127.0	449	50.1	130.0	405.6	3.0 – 3797.7	386	74.2	226.8	723.6	0 – 6711.0
Intestinal	125	28.3	58.4	118.3	2.0 – 835.1	196	483.0	838.5	1341.3	65.0 – 3680.1	234	63.6	149.4	461.8	3.3 – 3797.7	217	111.8	275.2	788.5	0 – 6711.0
Diffuse	85	22.5	48.9	113.3	2.1 – 2022.4	99	391.1	988.9	1504.3	5.8 – 3137.9	134	31.0	79.2	279.6	3.0 – 2510.6	93	21.5	61.9	354.0	0 – 4599.0
Mixed	17	15.9	58.5	133.1	7.3 – 209.7	24	549.2	1062.4	2127.6	180.4 – 2434.4	29	50.2	130.0	354.3	12.9 – 1150.3	26	59.4	225.6	620.2	15.1 – 2817.3
Unclassified	26	40.4	77.0	151.0	7.1 – 326.4	34	562.0	815.2	1211.0	65.1 – 4127.0	45	65.7	178.4	525.8	6.3 – 3746.8	44	165.7	337.4	1491.8	23.8 – 5089.8
Total Women	103	22.4	55.1	101.0	2.0 – 2022.4	127	530.5	1045.7	1498.4	9.7 – 3137.9	162	43.2	129.5	421.1	4.3 –3797.7	130	52.0	161.5	706.5	0 – 6711.0
Total Men	150	27.7	58.2	125.3	3.2 – 835.1	226	478.0	821.2	1338.4	5.8 – 4127.0	280	55.6	128.2	377.7	3.0 – 3071.1	250	90.4	238.9	736.3	0 – 5089.8

### TAN density as a function of tumor type

Next, we correlated TAN densities with the histological phenotype according to Laurén. With regard to the individual tumor type (i.e. intestinal, diffuse, mixed, and unclassified), again, median TAN densities varied between the four different compartments (Table 1). In connection with the individual compartments, no significant differences were found in the mucosa and tumor surface. However, TAN densities in the tumor center and invasion front differed significantly between diffuse and non-diffuse type, with GCs having the lowest median number of TANs in diffuse type GC (tumor center: *p* < 0.001; invasion front: *p* < 0.001). These data provide evidence that diffuse type GC and non-diffuse type GCs may differ in their ability to recruit TANs into the tumor center and invasion front.

### TAN density as a function of sex

**Fig. 2:**
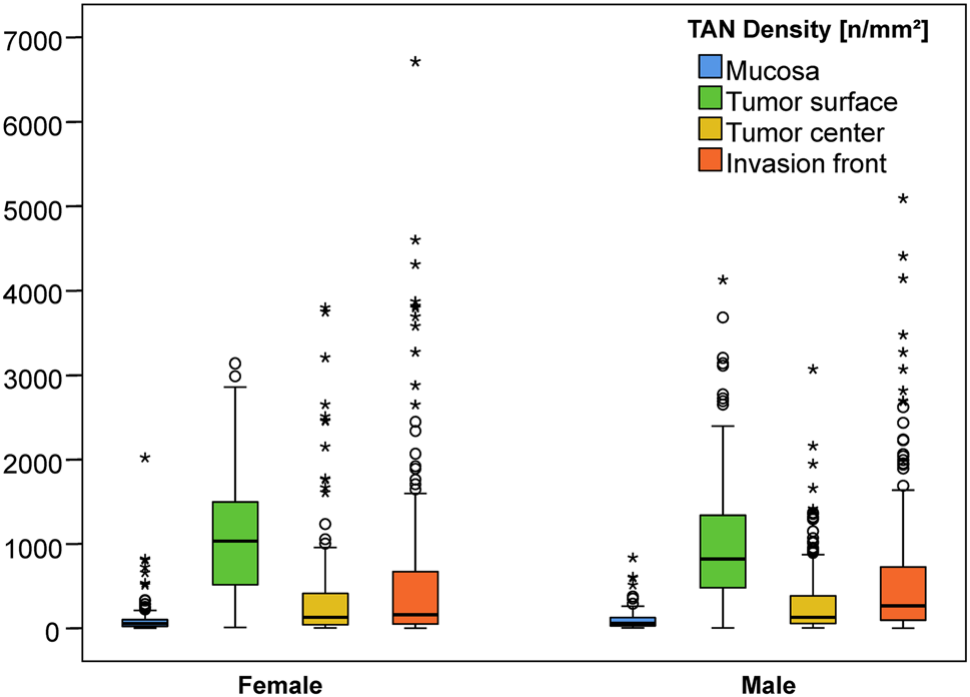
Boxplots: Densities of myeloperoxidase-immunoreactive tumor-associated neutrophils split by sex in the tumor compartments: nonneoplastic peritumoral mucosa (blue), tumor surface (green), tumor center (yellow) and invasion front (orange)

Thereafter, we correlated TAN density with sex. Again, differences were found in the overall distribution of TANs in the four different compartments (Table 1, Fig. 2). However, it was interesting to note that the increase of TAN density from the tumor center to the invasion front was greater in men (128.2 vs. 238.9 TANs/mm^2^) compared with women (129.5 vs. 161.5 TANs/mm^2^). TANs may contribute to GC biology in both men and women but show subtle spatially related differences in median densities. Clinicopathological features closely linked to sex were: patients’ age, localization, Laurén phenotype, EBV- and MET status (Suppl. Table 1).

### Exploration of the tumor biological effect of TANs as a function of TAN density

The assessment of the median values of TANs showed that densities vary, and we next sought to test the hypothesis that TAN density in each compartment (tumor surface, tumor center and invasion front) may correlate with clinicopathological patient characteristics. Using an explorative approach, we first categorized TAN density into “low” and “high” by splitting at the median (Suppl. Table 2). This categorization showed that TAN density correlated with different clinicopathological patient characteristics, e.g., tumor type according to Laurén and MSI status (Suppl. Table 2). However, given the large range of TAN density in GC (0–6711.0 TANs/mm^2^), we considered that dichotomization of the densities at the median may underestimate the effect of “lower” TAN counts. Therefore, we categorized TAN densities into four quartiles (Q1, Q2, Q3, Q4). This largely showed similar results (Suppl. Table 3), and we then hypothesized that, similar to reference values in whole blood counts, TAN density may have “a (near) physiological range” corresponding to the lowest quartile and a “pathological range” corresponding to the remaining quartiles. Hence, we next dichotomized the densities into a TAN-low group and a TAN-high group by comparing Q1 with Q2-Q4, respectively (Table 2). This showed that TAN density in the tumor center is related to Laurén phenotype (significant after multiple testing correction), tumor grade (significant after multiple testing correction), and EBV and MSI status. At the invasion front, TAN density is related to patient sex, Laurén phenotype (significant after multiple testing correction), tumor grade, T-category, EBV, MSI (significant after multiple testing correction), and HER2 status and patient survival (Table 2). Collectively, these data show that TANs are biologically relevant to tumors in GC and that the effect can already be demonstrated when the cutoff is set below the median values (i.e., Q1 vs. Q2–Q4).

**Table 2:** Correlation of the densities of myeloperoxidase-immunoreactive tumor-associated neutrophils dichotomized into quartile 1 vs. quartile 2–4 with clinicopathological patient characteristics. (1) Fisher’s exact test. (2) Kendall’s tau test. (3) Log-rank test

			Total		Mucosa				Tumor surface				Tumor center				Invasion front			
					Q_1_		Q_234_		Q_1_		Q_234_		Q_1_		Q_234_		Q_1_		Q_234_	
			Valid/missing value		Valid				Valid				Valid				Valid			
			*n/n*	(%)	*n*	(%)	*n*	(%)	*n*	(%)	*n*	(%)	*n*	(%)	*n* n	(%)	*n* n	(%)	*n*	(%)
Gender	*n*	*p* value^(1)^	449/0		257			0.242	358			0.899	449			0.071	386			0.013
Female			164	(36.5)	30	(28.8)	74	(71.2)	31	(24.2)	97	(75.8)	49	(29.9)	115	(70.1)	43	(32.8)	88	(67.2)
Male			285	(63.5)	34	(22.2)	119	(77.8)	58	(25.2)	172	(74.8)	63	(22.1)	222	(77.9)	53	(20.8)	202	(79.2)
Age group	*n*	*p* value^(1)^	449/0		257			0.774	358			0.142	449			0.744	386			0.906
< 68 years			221	(49.2)	32	(24.1)	101	(75.9)	49	(28.7)	122	(71.3)	57	(25.8)	164	(74.2)	46	(24.5)	142	(75.5)
≥ 68 years			228	(50.8)	32	(25.8)	92	(74.2)	40	(21.4)	147	(78.6)	55	(24.1)	173	(75.9)	50	(25.3)	148	(74.7)
Localisation	*n*	*p* value^(1)^	440/9		250			1.0	350			0.794	440			0.639	378			0.063
Proximal stomach			143	(31.8)	20	(26.0)	57	(74.0)	30	(26.1)	85	(73.9)	38	(26.6)	105	(73.4)	25	(19.5)	103	(80.5)
Distal stomach			297	(66.1)	44	(25.4)	129	(74.6)	58	(24.7)	177	(75.3)	72	(24.2)	225	(75.8)	71	(28.4)	179	(71.6)
Laurén type	*n*	*p* value^(1)^	449/0		257			0.516	358			0.493	449			<0.001*	386			<0.001*
Intestinal			239	(53.2)	29	(22.7)	99	(77.3)	50	(25.0)	150	(75.0)	42	(17.6)	197	(82.4)	34	(15.3)	188	(84.7)
Diffuse			136	(30.3)	24	(27.9)	62	(72.1)	28	(28.0)	72	(72.0)	54	(39.7)	82	(60.3)	51	(54.3)	43	(45.7)
Mixed			29	(6.5)	6	(35.3)	11	(64.7)	3	(12.5)	21	(87.5)	7	(24.1)	22	(75.9)	7	(26.9)	19	(73.1)
Unclassified			45	(10.0)	5	(19.2)	21	(80.8)	8	(23.5)	26	(76.5)	9	(20.0)	36	(80.0)	4	(9.1)	40	(90.9)
T-category	*n*	*p* value^(2)^	449/0		257			0.127	358			0.082	449			0.063	386			0.017
T1a/b			52	(11.6)	7	(17.9)	32	(82.1)	9	(20.5)	35	(79.5)	7	(13.5)	45	(86.5)	8	(15.7)	43	(84.3)
T2			53	(11.8)	10	(27.8)	26	(72.2)	10	(20.8)	38	(79.2)	12	(22.6)	41	(77.4)	8	(15.7)	43	(84.3)
T3			185	(41.2)	21	(20.8)	80	(79.2)	34	(22.8)	115	(77.2)	48	(25.9)	137	(74.1)	43	(26.7)	118	(73.3)
T4a/b			159	(35.4)	26	(32.1)	55	(67.9)	36	(30.8)	81	(69.2)	45	(28.3)	114	(71.7)	37	(30.1)	86	(69.9)
N-category	*n*	*p* value^(2)^	449/0		257			0.011	358			0.468	449			0.174	386			0.751
N0			126	(28.1)	16	(19.5)	66	(80.5)	23	(22.1)	81	(77.9)	26	(20.6)	100	(79.4)	25	(21.2)	93	(78.8)
N1			67	(14.9)	9	(20.0)	36	(80.0)	16	(29.6)	38	(70.4)	18	(26.9)	49	(73.1)	16	(29.6)	38	(70.4)
N2			78	(17.4)	7	(16.3)	36	(83.7)	11	(19.3)	46	(80.7)	18	(23.1)	60	(76.9)	20	(30.3)	46	(69.7)
N3a/b			178	(39.6)	32	(36.8)	55	(63.2)	39	(27.3)	104	(72.7)	50	(28.1)	128	(71.9)	35	(23.6)	113	(76.4)
M-category	*n*	*p* value^(1)^	449/0		257			0.575	358			0.871	449			0.486	386			0.202
M0			364	(81.1)	51	(24.2)	160	(75.8)	73	(24.6)	224	(75.4)	88	(24.2)	276	(75.8)	76	(23.5)	247	(76.5)
M1			85	(18.9)	13	(28.3)	33	(71.7)	16	(26.2)	45	(73.8)	24	(28.2)	61	(71.8)	20	(31.7)	43	(68.3)
UICC stage 8th eds.	*n*	*p* value^(2)^	449/0		257			0.195	358			0.482	449			0.118	386			0.115
IA/B			74	(16.5)	11	(20.8)	42	(79.2)	13	(20.3)	51	(79.7)	12	(16.2)	62	(83.8)	13	(18.1)	59	(81.9)
IIA/B			101	(22.5)	13	(20.3)	51	(79.7)	21	(25.6)	61	(74.4)	26	(25.7)	75	(74.3)	23	(25.6)	67	(74.4)
IIIA/B/C			189	(42.1)	27	(28.7)	67	(71.3)	39	(25.8)	112	(74.2)	50	(26.5)	139	(73.5)	40	(24.8)	121	(75.2)
IV			85	(18.9)	13	(28.3)	33	(71.7)	16	(26.2)	45	(73.8)	24	(28.2)	61	(71.8)	20	(31.7)	43	(68.3)

			Total		Mucosa				Tumor surface				Tumor center				Invasion front			
					Q_1_		Q_234_		Q_1_		Q_234_		Q_1_		Q_234_		Q_1_		Q_234_	
			Valid/missing value		Valid				Valid				Valid				Valid			
			*n/n*	(%)	*n*	(%)	*n*	(%)	*n*	(%)	*n*	(%)	*n*	(%)	*n*	(%)	*n*	(%)	*n*	(%)
Lymph node ratio	*n*	*p* value^(1)^	449/0		257			0.006	358			0.328	449			0.326	386			0.906
< 0.189			220	(49.0)	26	(18.2)	117	(81.8)	40	(22.3)	139	(77.7)	50	(22.7)	170	(77.3)	49	(25.3)	145	(74.7)
≥ 0.189			229	(51.0)	38	(33.3)	76	(66.7)	49	(27.4)	130	(72.6)	62	(27.1)	167	(72.9)	47	(24.5)	145	(75.5)
L-category	*n*	*p* value^(1)^	423/26		242			0.106	338			0.528	423			0.824	365			0.904
L0			202	(45.0)	26	(20.8)	99	(79.2)	37	(22.8)	125	(77.2)	50	(24.8)	152	(75.2)	44	(24.9)	133	(75.1)
L1			221	(49.2)	35	(29.9)	82	(70.1)	46	(26.1)	130	(73.9)	57	(25.8)	164	(74.2)	48	(25.5)	140	(74.5)
V-category	*n*	*p* value^(1)^	422/27		241			0.146	336			0.409	422			0.860	365			1.0
V0			374	(83.3)	58	(26.7)	159	(73.3)	70	(23.3)	230	(76.7)	95	(25.4)	279	(74.6)	82	(25.4)	241	(74.6)
V1			48	(10.7)	3	(12.5)	21	(87.5)	11	(30.6)	25	(69.4)	11	(22.9)	37	(77.1)	11	(26.2)	31	(73.8)
Tumor grade	*n*	*p* value^(1)^	447/2		256			0.395	358			0.577	447			0.001*	384			0.008
G1/2			108	(24.1)	12	(19.7)	49	(80.3)	25	(27.2)	67	(72.8)	14	(13.0)	94	(87.0)	16	(15.2)	89	(84.8)
G3/G4			339	(75.5)	51	(26.2)	144	(73.8)	64	(24.1)	202	(75.9)	96	(28.3)	243	(71.7)	79	(28.3)	200	(71.7)
Resection status	*n*	*p* value^(1)^	436/13		248			0.109	348			0.694	436			0.503	374			0.717
R0			382	(85.1)	51	(23.3)	168	(76.7)	78	(25.2)	231	(74.8)	96	(25.1)	286	(74.9)	83	(25.2)	247	(74.8)
R1/R2			54	(12.0)	11	(37.9)	18	(62.1)	8	(20.5)	31	(79.5)	11	(20.4)	43	(79.6)	12	(27.3)	32	(72.7)
H. pylori status	*n*	*p* value^(1)^	379/70		231			0.548	302			0.723	379			0.106	332			0.612
Negative			320	(71.3)	50	(26.2)	141	(73.8)	57	(23.0)	191	(77.0)	88	(27.5)	232	(72.5)	71	(25.6)	206	(74.4)
Positive			59	(13.1)	8	(20.0)	32	(80.0)	14	(25.9)	40	(74.1)	10	(16.9)	49	(83.1)	12	(21.8)	43	(78.2)
EBV status	*n*	*p* value^(1)^	438/11		250			0.368	351			0.397	438			0.022	375			0.009
Negative			416	(92.7)	61	(26.0)	174	(74.0)	79	(23.7)	254	(76.3)	108	(26.0)	308	(74.0)	93	(26.1)	264	(73.9)
Positive			22	(4.9)	2	(13.3)	13	(86.7)	6	(33.3)	12	(66.7)	1	(4.5)	21	(95.5)	0	(0.0)	18	(100.0)
MSI status	*n*	p-value^(1)^	437/12		249			0.009	352			0.813	437			0.035	375			0.002*
MSS			405	(90.2)	63	(27.3)	168	(72.7)	79	(24.2)	247	(75.8)	105	(25.9)	300	(74.1)	92	(26.7)	252	(73.3)
MSI			32	(7.1)	0	(0.0)	18	(100.0)	7	(26.9)	19	(73.1)	3	(9.4)	29	(90.6)	1	(3.2)	30	(96.8)
HER2 status	*n*	*p* value^(1)^	412/37		232			0.788	329			0.082	412			0.066	352			0.006
Negative			376	(83.7)	53	(25.0)	159	(75.0)	68	(22.9)	229	(77.1)	98	(26.1)	278	(73.9)	87	(27.4)	231	(72.6)
Positive			36	(8.0)	4	(20.0)	16	(80.0)	12	(37.5)	20	(62.5)	4	(11.1)	32	(88.9)	2	(5.9)	32	(94.1)
MET status	*n*	*p* value^(1)^	440/9		253			0.408	351			0.808	440			0.665	378			1.0
Negative			409	(91.1)	58	(24.7)	177	(75.3)	82	(25.1)	245	(74.9)	102	(24.9)	307	(75.1)	86	(24.3)	268	(75.7)
Positive			31	(6.9)	6	(33.3)	12	(66.7)	5	(20.8)	19	(79.2)	6	(19.4)	25	(80.6)	6	(25.0)	18	(75.0)
OSS [months]		*p* value^(3)^						0.052				0.134				0.142				0.017
Total/Events/Censored			433/341/92		60/50/10		186/139/47		88/70/18		258/204/54		109/88/21		324/253/71		92/75/17		278/207/71	
Median Survival			15.0 ± 1.2		14.0 ± 2.6		18.8 ± 2.3		13.2 ± 2.1		16.7 ± 1.4		12.9 ± 1.6		16.0 ± 1.4		13.6 ± 2.2		16.7 ± 1.9	
95% C.I.			12.7–17.3		8.9–19.1		14.2–23.3		9.1–17.4		13.9–19.5		9.7–16.2		13.2–18.8		9.3–17.9		13.0–20.4	
TSS [months]		*p* value^(3)^						0.073				0.156				0.137				0.013
Total/Events/Censored			405/280/125		49/35/14		179/111/68		84/60/24		238/161/77		103/75/28		302/205/97		87/65/22		260/164/96	
Median Survival			16.7 ± 1.5		13.2 ± 3.8		22.6 ± 2.5		14.6 ± 3.5		18.0 ± 2.2		14.2 ± 2.0		17.9 ± 2.6		14.7 ± 2.9		19.6 ± 3.8	
95% C.I.			13.7–19.7		5.7–20.7		17.6–27.6		7.8–21.4		13.6–22.3		10.2–18.1		12.8–23.1		9.0–20.4		12.2–27.0	

(1) Fisher’s exact test, (2) Kendall’s tau test, (3) Log-rank test, * Significant after FDR-Control

### Prognostic significance of TAN densities

Finally, we were interested in exploring the correlation between TAN density and patient survival (Table 2; Suppl. Tables 2 and 3). Dichotomization of the patient cohort at the median (Suppl. Table 2) and at the four quartiles (Suppl. Table 3) showed no differences in tumor-specific survival. However, when TAN density was dichotomized into Q1 (low) and Q2–4 (high), patients with high TAN densities at the invasion front had a much more favorable overall survival (OS; median survival 13.6 ± 2.2 months TAN-low vs. 16.7 ± 1.9 months TAN-high; *p* = 0.017) and tumor-specific survival (TSS; median survival 14.7 ± 2.9 months TAN-low vs. 19.6 ± 3.8 months TAN-high; *p* = 0.013) compared to patients with low TAN densities. However, after multiple testing correction these results were no longer significant.

**Fig. 3:**
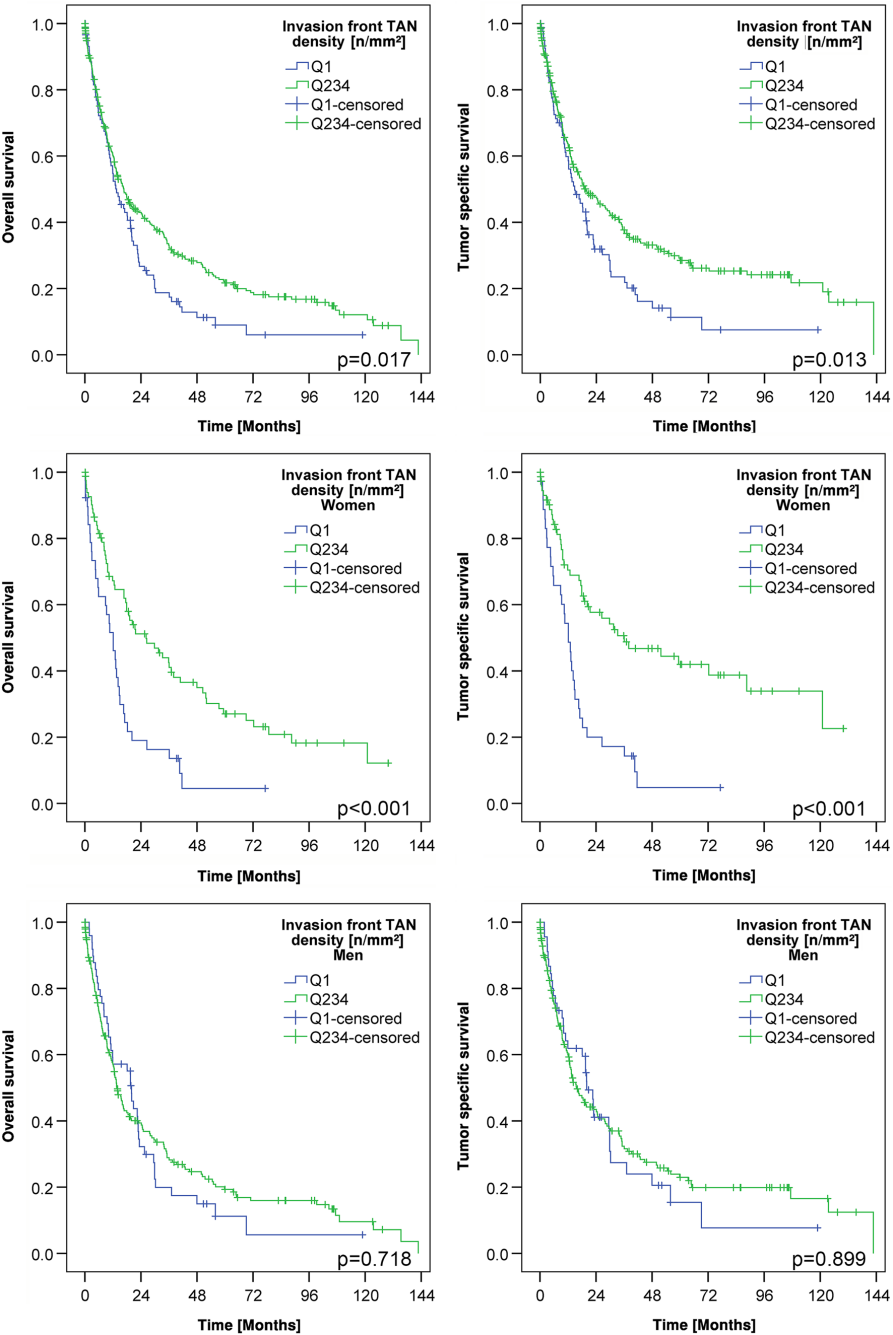
Patients’ survival: Kaplan–Meier curves of the entire cohort depicting patients’ overall and tumor-specific survival according to the densities of myeloperoxidase-immunoreactive tumor-associated neutrophils in the invasion front (dichotomized into quartile 1 vs. quartile 2–4; upper panel). Women with a low density of myeloperoxidase-immunoreactive tumor-associated neutrophils had a poorer survival compared to those with a high density of myeloperoxidase-immunoreactive tumor-associated neutrophils (middle panel). This effect could not be shown in men (lower panel). All *p* values shown in the graph were obtained by log-rank test

**Fig. 4:**
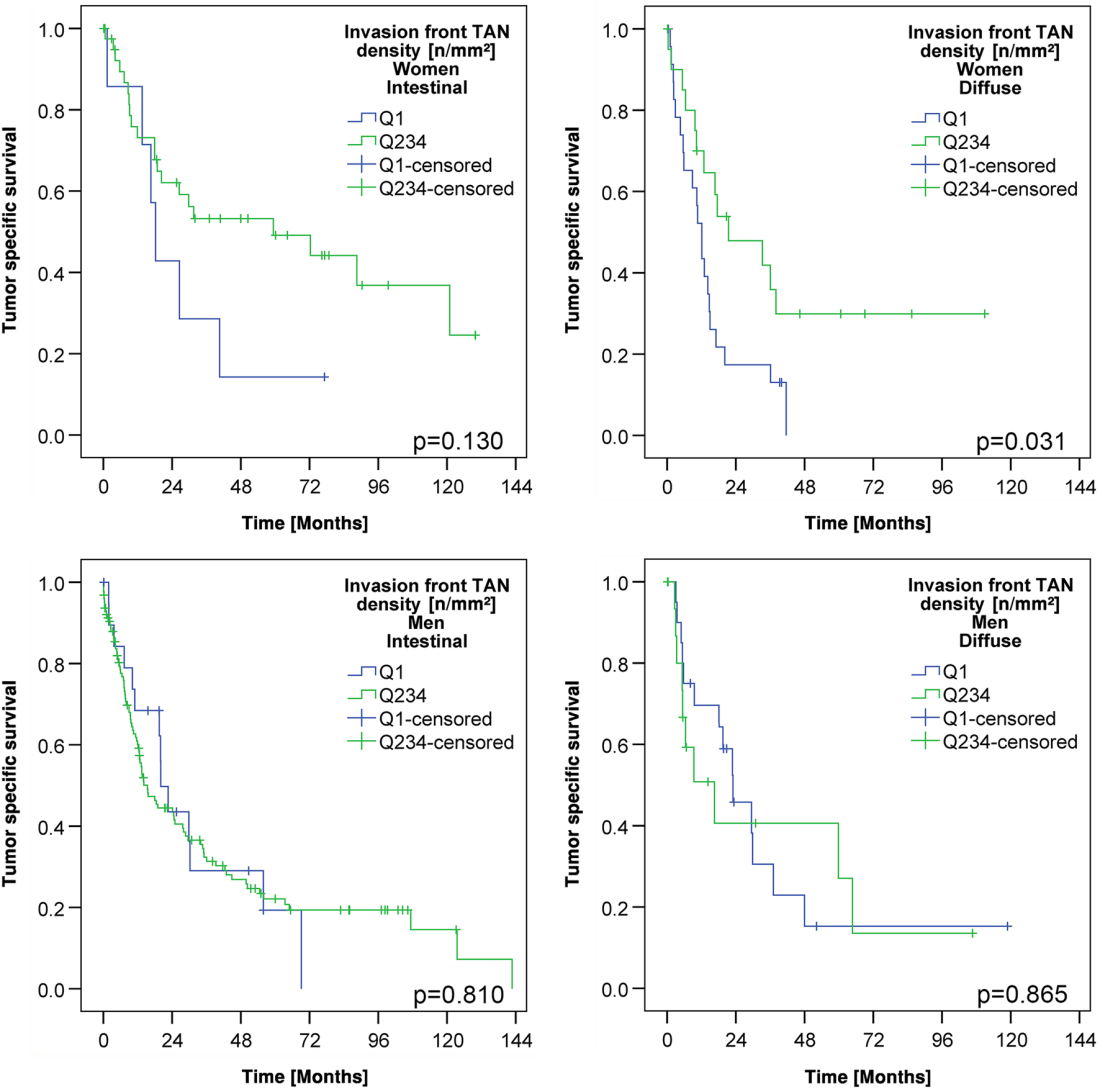
Patients’ survival in intestinal and diffuse phenotype: Kaplan–Meier curves showing the association between the densities of myeloperoxidase-immunoreactive tumor-associated neutrophils in the invasion front (dichotomized into quartile 1 vs. quartile 2–4) and patients’ tumor-specific survival. Independent of the Laurén phenotype, women with a low density of myeloperoxidase-immunoreactive tumor-associated neutrophils had a poorer survival compared to those with a high density of myeloperoxidase-immunoreactive tumor-associated neutrophils (*upper panel*). This effect could not be shown in men (lower panel). All *p* values shown in the graph were obtained by log-rank test

Since we noticed sex-specific differences in the TAN density at the invasion front (Table 2), we next explored patient survival separately for men and women. This finally showed that TAN density had no impact on patient survival in men, while it was highly significantly correlated with patient outcome in women. Women with high TAN densities (Q2–Q4) in the tumor center had a better OS (median survival 12.9 ± 1.6 months TAN-low vs. 17.9 ± 2.4 months TAN-high; p = 0.079) and a significantly better TSS (median survival 12.8 ± 1.5 months TAN-low vs. 18.8 ± 3.7 months TAN-high; *p* < 0.026) compared with women with low TAN densities. Women with high TAN densities (Q2–Q4) at the invasion front had also a significantly better OS (median survival 12.1 ± 2.2 months TAN-low vs. 26.3 ± 7.1 months TAN-high; *p* < 0.001) and TSS (median survival 12.1 ± 1.7 months TAN-low vs. 36.1 ± 14.0 months TAN-high; *p* < 0.001) compared with women with low TAN densities (Q1; Fig. 3; Suppl. Fig. 2). This effect was not related to tumor type according to Laurén (Fig. 4) and there was also no general difference of TSS between men and women in our cohort (Suppl. Fig. 3).

### Univariate and multivariate survival analysis

The entire GC collective showed a median OS of 15.0 months and a median TSS of 16.7 months. Patient prognosis significantly depended on the Laurén phenotype, T-, N-, M-, L-, V- , and R-category, UICC stage, grade, lymph node ratio, MSI status, MET status (data not shown), and TAN density at the invasion front (*p* = 0.013). TSS of women depended on Laurén phenotype, T-, N-, M-, L-, V-, and R-category, UICC stage, grade, lymph node ratio, MET status, and TAN density at tumor center and invasion front. On multivariate analysis, TAN density at the invasion front (HR 2.77; 95% CI 1.64–4.77), R-, L-, and N-category turned out to be significant and independent prognosticators of women’s tumor-specific survival (Table 3; Suppl. Fig. 1).

**Table 3:** Univariate and multivariate analysis of tumor specific survival in female gastric cancer patients

		Tumor specific univariate analysis				Multivariate analysis			
		Total/events/censored	Median survival	95% CI	*p* (log-rank test)		HR	95% CI	*p* value
T-category	T1a/b	19/3/16	n.c.	n.c.	<0.001				
	T2	15/7/8	51.7 ± 23.9	4.9–98.6					
	T3	55/38/17	20.3 ± 8.3	4.1–36.5					
	T4a/b	58/50/8	8.9 ± 2.1	4.8–13.0					
M-category	M0	110/65/45	26.1 ± 6.3	13.7–38.5	<0.001				
	M1	37/33/4	7.3 ± 2.4	2.6–12.0					
N-category	N0	46/17/29	88.5 ± 22.9	43.7–133.4	<0.001				0.003
	N1	25/16/9	17.1 ± 3.3	10.5–23.6		N1 vs. N0	2.12	0.88–5.09	0.094
	N2	20/15/5	18.2 ± 4.5	9.4–27.0		N2 vs. N0	2.35	0.97–5.68	0.058
	N3a/b	56/50/6	9.3 ± 1.5	6.3–12.3		N3a/b vs. N0	4.07	1.95–8.48	<0.001
L-category	L0	69/31/38	44.6 ± 16.1	13.1–76.2	<0.001	L1 vs. L0	2.10	1.14–3.85	0.017
	L1	73/63/10	10.6 ± 2.7	5.3–16.0					
V-category	V0	127/82/45	17.5 ± 2.2	13.1–21.9	0.030				
	V1	16/13/3	11.9 ± 5.8	0.5–23.3					
Resection status	R0	130/81/49	18.2 ± 3.4	11.6–24.8	<0.001	R1 vs. R0	6.73	3.14–14.40	<0.001
	R1	17/17/0	5.1 ± 2.3	0.5–9.7					
Tumor grade	G1/2	26/10/16	88.5 ± 21.5	46.4–130.6	<0.001				
	G3/4	121/88/33	14.2 ± 1.9	10.4–17.9					
LN-Ratio	<0.189	79/38/41	40.6 ± 13.9	13.2–67.9	<0.001				
	≥0.189	68/60/8	9.7 ± 0.8	8.1–11.3					
Laurén type	Intestinal	56/31/25	29.8 ± 13.0	4.3–55.3	0.004				
	Diffuse	72/55/17	14.2 ± 1.6	11.0–17.4					
	Mixed	6/5/1	4.5 ± 0.9	2.6–6.4					
	Unclassified	13/7/6	24.4 ± 13.2	0–50.2					
MSI status	MSS	134/93/41	15.5 ± 1.7	12.1–18.9	0.060				
	MSI	12/5/7	51.7 ± n.c.	n.c.					
MET status	Negative	139/92/47	17.5 ± 2.2	13.3–21.7	0.005				
	Positive	5/5/0	3.6 ± 1.3	1.1–6.2					
TAN density in tumor	Q1	46/36/10	12.8 ± 1.5	9.9–15.8	0.026				
center	Q2–Q4	101/62/39	18.8 ± 3.7	11.5–26.0					
TAN density at invasion front	Q1	41/32/9	12.1 ± 1.7	8.9–15.4	<0.001	Q1 vs. Q2-Q4	2.77	1.64–4.77	<0.001
	Q2–Q4	75/39/36	36.1 ± 14.0	8.6–63.5					

## Discussion

Sexual dimorphism in immune response capacity relates to differences in immune surveillance competence between men and women, which may also contribute to the sex effect observed in malignant tumors (Dorak and Karpuzoglu 2012). Our study confirms a previous finding from Caruso et al. (Caruso et al. 2002) of a sex-specific difference in the tumor biological effect of TANs in GC biology by using an independent European patient cohort. The TAN density at the invasion front (HR 2.77; 95% CI 1.64–4.77) of women with GC was an independent predictor of favorable patient outcome and even surpassed MSI status (Table 3). Interestingly, studies on TANs in Asian cohorts were unable to find any sex-specific differences (Fu et al. 2016; Huang et al. 2018; Li et al. 2017; Zhang et al. 2018; Zhao et al. 2012). However, tumor immunity signatures differ significantly between Asian and non-Asian GC patients, including markers of TANs (i.e., CD66b) and may contribute to differences in clinical outcome (Lin et al. 2015).

Apart from ethnicity, sex has to be considered in regard to the exploitation of immune surveillance and evasion in GC biology, particularly in patients of Western descent. Important immunity genes are carried on the X chromosome, and sex chromosomes are believed to have a higher impact on innate immunity than sex hormones. However, sex hormones regulate the expression of many genes. Female hormones delay neutrophil apoptosis and, depending on the menstrual cycle, modulate their chemotaxis and recruitment (Jaillon et al. 2019). Palli et al. reported that women with a longer fertility duration or a later menopause showed a lower risk of developing GC (Palli et al. 1994). Testosterone, on the other hand, is known to have immunosuppressive effects and causes increased neutrophil activation in noninfectious inflammatory states, and androgen ablation reduces neutrophils (Jaillon et al. 2019). Neutrophils also show sexual differences in appearance (Chatterjee 2014). Spitzer et al. found that their phagocytic response is higher and more resistant to anesthesia and surgery in reproductive female versus male rats (Spitzer and Zhang 1996).

TANs belong to the cellular component of TIME, which had been categorized into three classes (Binnewies et al. 2018): (a) the infiltrated-excluded TIME is characterized by the exclusion of cytotoxic T-cells from the tumor core; (b) the infiltrated-inflamed TIME is considered to be an immunologically ‘hot’ tumor and is characterized by high infiltration of CTLs expressing programmed cell death protein 1 (PD-1) and leukocytes and tumor cells expressing the immune-dampening PD-1 ligand PD-L1; and (c) a subclass of infiltrated-inflamed TIME, i.e., TLS-TIME, includes tertiary lymphoid structures and lymphoid aggregates whose cellular composition is similar to that found in lymph nodes (Binnewies et al. 2018). TIME is a function of both tumor genotype/phenotype and immunological composition. This might explain the striking differences in TAN densities between diffuse and non-diffuse type GC (Table 1). Recently, a four-tiered molecular classification of GC was proposed, i.e., Epstein-Barr-virus (EBV)-positive, microsatellite instable (MSI), chromosomal instable and genomically stable GCs (Cancer Genome Atlas Research 2014). The diffuse phenotype is often genomically stable and enriched for mutations in *CDH1*, *RHOA* and various other cell adhesion molecules (Cancer Genome Atlas Research 2014). Our findings confirm observations made by Abe et al. (Abe et al. 2016), where TAN densities varied depending on the Laurén phenotype. In contrast, Fu et al. described different TAN distribution patterns in intestinal and diffuse cancer types, but no differences in TAN densities (Fu et al. 2016). Nonetheless, all these data support the notion that the genotype/phenotype of GC impacts the cellular composition of TIME, including TANs and that the diffuse type GC is characterized by a low density or absence of TANs.

In our cohort, TAN densities were also related to EBV and MSI status, both in tumor center and invasion front. EBV- and MSI-associated GCs are known to induce a strong inflammatory response, which apparently also includes recruitment of TAN, as has also been shown recently by Abe et al. for EBV-GC (Abe et al. 2016). MSI was believed to primarily affect the adaptive immune system. However, innate immunity may also be effective in MSI-GC, and further studies on this topic are warranted.

Neither the tumor nor the TIME is static during tumor progression. This might explain the steady decline of the value of TAN-high (Q2–Q4) at the invasion front. While the number of cases with high TAN density (Q2–Q4) at the invasion front was 84.3% in T1-tumors, it declined significantly to 69.9% in T4-tumors (Table 2). Similarly, we previously observed significant differences in the expression of PD-L1 and V-domain Ig suppressor of T cell activation (VISTA) in relation to the T-category (Böger et al. 2016, 2017). Thus, while GC progresses locally, TIME changes with decreasing TAN density, further supporting a putative tumor-protective role of TANs in GC biology. Interestingly, TAN densities were higher in men compared with women, which leads to the conjecture that TANs seem to have different phenotypes in GC: women might be able to activate a more effective N1-TAN phenotype. However, there is currently no validated biomarker for the characterization of TAN phenotypes in tissue sections, and other mechanisms might be effective.

Different from previous attempts on the exploitation of TANs in GC using selected high-power fields (Caruso et al. 2002; Li et al. 2017; Zhao et al. 2012) or tissue micro arrays (Huang et al. 2018), we were specifically interested in the spatial distribution of TANs in GC by using digital image analysis on whole tissue sections and well-defined tumor areas, limiting the risk of observer bias. We found significant differences in the TAN densities with regard to their spatial distribution (tumor surface vs. tumor center vs. invasion front). We noticed a specific enrichment at the invasion front, which was also noticed by Li et al. (Li et al. 2017). Previously, we already collected ample evidence for the intratumoral heterogeneity of GC, which now also applies to TANs. With regard to the immune evasion mechanism of GC, we previously found different PD-L1 expression patterns in GC (Böger et al. 2016). MSI-GCs were mainly PD-L1-positive at the interface between neoplastic and nonneoplastic tissue, especially in areas of “pushing borders”, pointing towards the invasion front as being a specific and tumor-biologically important TIME microniche in GC.

Summing up, our retrospective study on a large European cohort shows that TANs are a specific cellular component of the TIME with tumor-biological significance. TANs in GC are related to the genotype/phenotype, show unique spatial (tumor surface vs. tumor center vs. invasion front) and “temporal” (T-category) distribution patterns and are even an independent predictor of patient outcome in women. Therefore, our study provides evidence of sexual dimorphism in the immune response capacity to GC, which should be taken into account in future studies and clinical trials.

### Study limitations

None of our patients received perioperative or neoadjuvant chemotherapy, and no comment can be made on the effect of chemotherapy on TANs in GC. We also did not study the suitability of endoscopic biopsies to asses TAN densities. However, since the invasion front is rarely captured by biopsies, the assessment of TAN status is prone to a sampling error. Due to the retrospective and observational character of our study using only formalin-fixed and paraffin-embedded tissue samples, we were unable to provide any functional data. However, our extensive morphological and statistical analyses may help to generate hypotheses for future experimental studies on the tumor-biological mechanisms of TANs in GC.

## Electronic supplementary material

Below is the link to the electronic supplementary material.

Supplemental Fig. 1 CONSORT diagram illustrating study design, search (inclusion) criteria and exclusion criteria. (PDF 14 kb)

Supplemental Fig. 2 Kaplan–Meier curves depicting patients’ overall and tumor-specific survival according to the densities of myeloperoxidase-immunoreactive tumor-associated neutrophils in the invasion front divided into quartiles. P-value shown in the graph was obtained by log-rank test. (TIFF 2159 kb)

Supplemental Fig. 3 Kaplan–Meier curve showing no association between sex and tumor-specific survival. P-value shown in the graph was obtained by log-rank test. (TIFF 196 kb)

Supplemental Table 1 Correlation of sex with clinicopathological patient characteristics. (1) Fisher’s exact test. (2) Kendall’s tau test. (3) Log-rank test. (XLSX 16 kb)

Supplemental Table 2 Correlation of the densities of myeloperoxidase-immunoreactive tumor-associated neutrophils dichotomized at the median into TAN-low and TAN-high groups with clinicopathological patient characteristics. (1) Fisher’s exact test. (2) Kendall’s tau test. (3) Log-rank test. (XLSX 23 kb)

Supplemental Table 3 Correlation of the densities of myeloperoxidase-immunoreactive tumor-associated neutrophils categorized by quartiles (Q1, Q2, Q3, Q4) with clinicopathological patient characteristics. (1) Fisher’s exact test. (2) Kendall’s tau test. (3) Log-rank test. (XLSX 31 kb)
