# Supplementary material for: Sexual dimorphism in gastric cancer: tumor-associated neutrophils predict patient outcome only for women
Source: J Cancer Res Clin Oncol. 2019 Nov 19;146(1):53–66. doi: 10.1007/s00432-019-03082-z (PMC6942031; doi:10.1007/s00432-019-03082-z)
Supplement: Supplementary file 1 — Supplemental Fig. 1 CONSORT diagram illustrating study design, search (inclusion) criteria and exclusion criteria. (PDF 14 kb) [file 432_2019_3082_MOESM1_ESM.pdf]

Retrospective cohort study

Primary search (inclusion) criteria: primary adenocarcinoma of the stomach or gastroesophageal junction confirmed histologically
Exclusion criteria: histology identified a tumor type other than adenocarcinoma or perioperative chemo- or radiotherapy; missing values in basic clinicopathological variables (i.e. sex, Laurén phenotype, T-, N- and M-category, or UICC stage) or less than two of the compartments (i.e. mucosa, tumor surface, tumor center, and invasion front) were excluded.

↓

Final study cohort
Total patients 1997-2009: 449
Total men: 285
Total women: 164

↓

Used for calculation of raw score values, correlations with gender, clinicopathological variables, overall survival and tumor-specific survival.
Used for univariate analysis.

↓ → Excluded for subgroup analysis: 295 male patients

Female patients only (total cases=164; cases available in analysis as shown in Table 3)

↓

Univariate and multivariate analysis of survival in female gastric cancer patients
Total cases : 164
Cases avaiable in analysis: 106 (69 with event; 37 censored)
Cases dropped: 58 (54 cases with missing values; 4 censored cases before the earliest event in a stratum)
