# Supplementary figures and images for: Sexual dimorphism in gastric cancer: tumor-associated neutrophils predict patient outcome only for women
Source: J Cancer Res Clin Oncol. 2019 Nov 19;146(1):53–66. doi: 10.1007/s00432-019-03082-z (PMC6942031; doi:10.1007/s00432-019-03082-z)

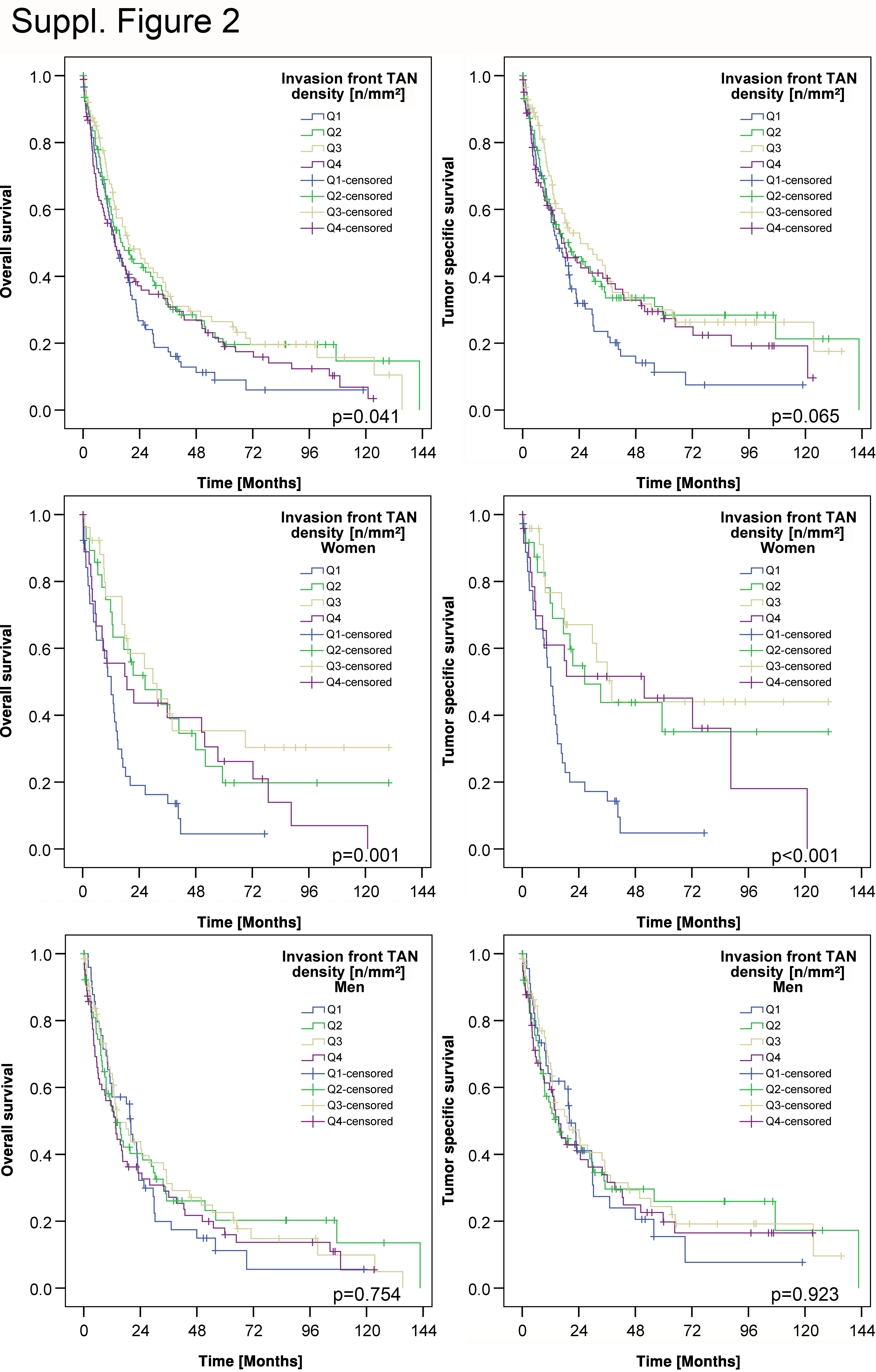

Supplement: Supplementary file 2 — Supplemental Fig. 2 Kaplan–Meier curves depicting patients’ overall and tumor-specific survival according to the densities of myeloperoxidase-immunoreactive tumor-associated neutrophils in the invasion front divided into quartiles. P-value shown in the graph was obtained by log-rank test. (TIFF 2159 kb) [file 432_2019_3082_MOESM2_ESM.tif]

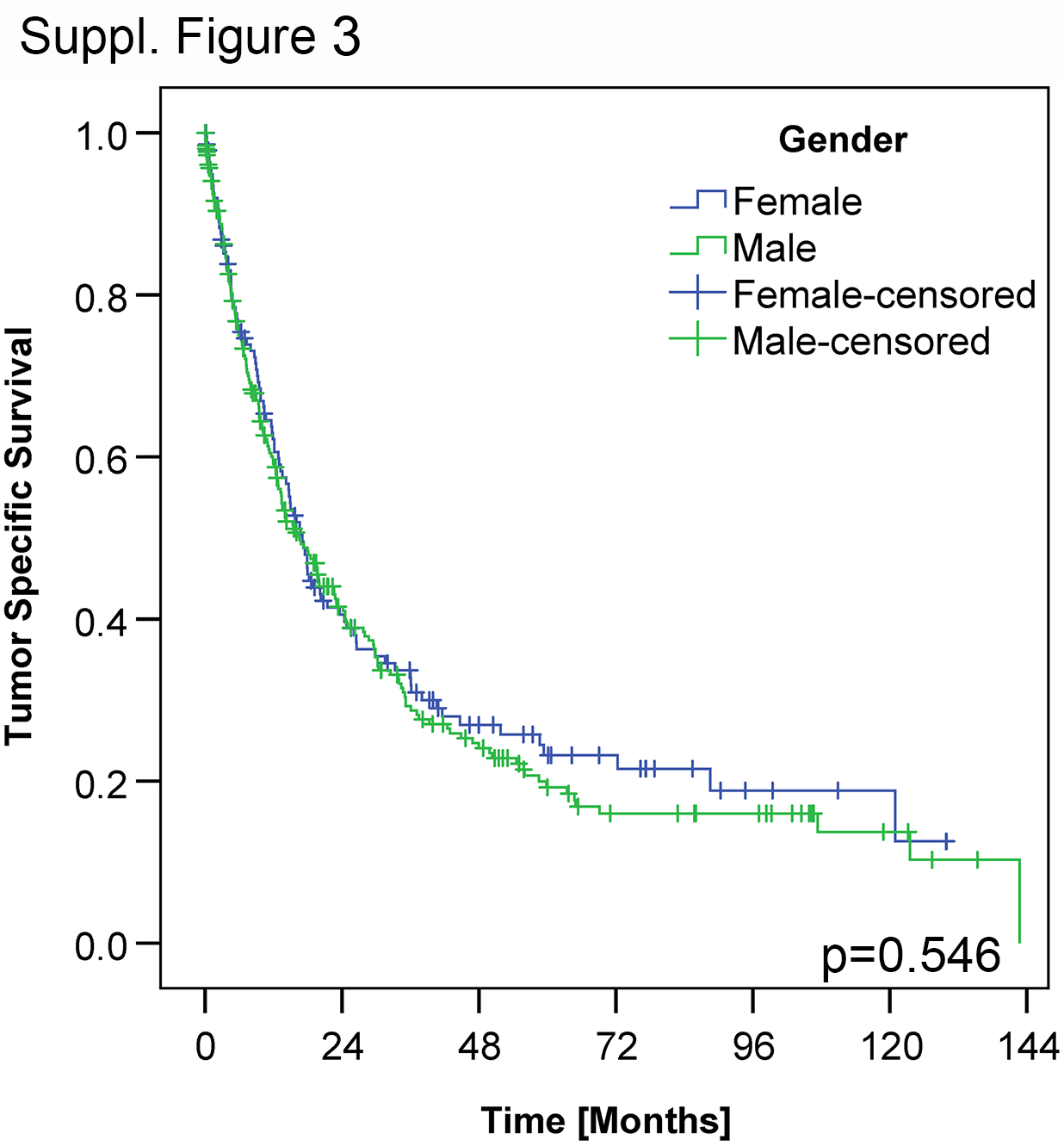

Supplement: Supplementary file 3 — Supplemental Fig. 3 Kaplan–Meier curve showing no association between sex and tumor-specific survival. P-value shown in the graph was obtained by log-rank test. (TIFF 196 kb) [file 432_2019_3082_MOESM3_ESM.tif]
